# Energy-dispersive X-ray stress analysis under geometric constraints: exploiting the material’s inherent anisotropy

**DOI:** 10.1107/S1600576723001759

**Published:** 2023-04-01

**Authors:** Christoph Genzel, Manuela Klaus, Nico Hempel, Thomas Nitschke-Pagel, Karen Pantleon

**Affiliations:** a Abteilung für Mikrostruktur- und Eigenspannungsanalyse, Helmholtz-Zentrum Berlin für Materialien und Energie, Berlin, Germany; b Institut für Füge- und Schweißtechnik, Technische Universität Braunschweig, Braunschweig, Germany; c Chair of Materials Engineering of Additive Manufacturing, Department of Materials Engineering, TUM School of Engineering and Design, Technical University of Munich, Munich, Germany; d Department of Civil and Mechanical Engineering, Technical University of Denmark, Kongens Lyngby, Denmark; Montanuniversität Leoben, Austria

**Keywords:** X-ray stress analysis, energy-dispersive diffraction, polycrystalline materials, single-crystal elastic anisotropy

## Abstract

Two data evaluation concepts for X-ray stress analysis based on energy-dispersive diffraction on polycrystalline materials with cubic crystal structure, almost random crystallographic texture and strong single-crystal elastic anisotropy are subjected to comparative assessment. The aim is the study of the residual stress state in hard-to-reach measurement points, for which the sin^2^ψ method is not applicable due to beam shadowing at larger sample tilting. This makes the approaches attractive for stress analysis in engineering parts with complex shapes, for example. Both approaches are based on the assumption of a biaxial stress state within the irradiated sample volume. They exploit in different ways the elastic anisotropy of individual crystallites acting at the microscopic scale and the anisotropy imposed on the material by the near-surface stress state at the macroscopic scale. They therefore complement each other, in terms of both their preconditions and their results. The first approach is based on the evaluation of strain differences, which makes it less sensitive to variations in the strain-free lattice parameter *a*_0_. Since it assumes a homogeneous stress state within the irradiated sample volume, it provides an average value of the in-plane stresses. The second approach exploits the sensitivity of the lattice strain to changes in *a*_0_. Consequently, it assumes a homogeneous chemical composition but provides a stress profile within the information depth. Experimental examples from different fields in materials science, namely shot peening of austenitic steel and *in situ* stress analysis during welding, are presented to demonstrate the suitability of the proposed methods.

## Abstract 2

The single-crystal elastic anisotropy and the anisotropy of the near-surface (residual) stress state of polycrystalline materials with random texture are exploited in energy-dispersive X-ray stress analysis to study samples under constrained measurement conditions.

## 1. Introduction

In most cases, X-ray stress analysis (XSA) of polycrystalline materials targets the investigation of the highly stressed near-surface zone. In the past few decades, numerous methods have been developed based on the measurement of the lattice strain for many different orientations with respect to a sample reference system. The strain data form the input for the fundamental equation of XSA (Stickforth, 1966 ▸; Evenschor & Hauk, 1975 ▸) to calculate the individual components of the (residual) stress tensor. An overview of the current status in this respect can be found, for example, in the textbooks by Noyan & Cohen (1987 ▸), Hauk (1997 ▸), Mittemeijer & Welzel (2013 ▸) and Spieß *et al.* (2019 ▸).

Almost all methods developed for near-surface stress analysis are based on measurements in reflection geometry and are limited in their applicability to freely accessible measuring points, and thus to simple specimen geometries with flat or convexly curved surfaces, in order to avoid beam shadowing during specimen tilting. Only the ‘strain scanning’ techniques (Withers & Webster, 2001 ▸) do not require sample tilting. However, if the measurements are performed in reflection mode (through surface strain scanning; Webster *et al.*, 1996 ▸), the results are mostly limited to lattice strain distributions perpendicular to the surface, since uncertainties in the strain-free lattice parameter lead to large errors in the stress calculation.

A review of the literature reveals that there is currently a lack of XSA methods that allow non-destructive analysis of the near-surface stress state on engineering parts and components with complex shapes, where the measurement points are difficult to access and prevent high specimen tilt. On the other hand, there is a high demand for such measurements, especially from industry, since failure-critical residual stresses are often expected at positions featuring strong concave curvatures, such as the tooth base of gears, sharp bends in formed components or the inner wall of boreholes. For this last case, we have recently presented an evaluation concept based on energy-dispersive (ED) diffraction (Genzel *et al.*, 2021 ▸) which takes into account the influence of the strongly curved surface but still allows the use of the sin^2^ψ method (Macherauch & Müller, 1961 ▸) due to the very small diffraction angles. However, for measurement points in notches and other positions with a very small opening angle, the sin^2^ψ-based measurement and evaluation strategies are no longer applicable.

The evaluation strategies introduced in this article aim to provide solutions for such problems. They are based on measurements performed in the ED diffraction mode exploiting high-energy synchrotron radiation or the *Brems­strahlung* emitted by high-flux laboratory X-ray sources. In addition to its large information depth, the ED method offers some further features that make it attractive for residual stress analysis. Bragg’s law in its ED form reads (Giessen & Gordon, 1968 ▸; Buras *et al.*, 1968 ▸)

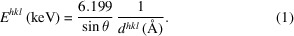

ED diffraction provides complete diffraction patterns with a multitude of diffraction lines *E* ^*hkl*^ under a fixed but freely selectable Bragg angle θ, which can be used to tune the diffraction-line position on the energy scale in order to adapt the information depth to different regions below the surface (Genzel & Klaus, 2017 ▸). Furthermore, each diffraction line *E* ^*hkl*^ originates from another average depth, which is an additional parameter available for a depth-resolved analysis. Pioneering work in the field of near-surface ED-XSA was carried out by Ruppersberg and co-workers. Their universal plot method (Ruppersberg *et al.*, 1989 ▸, 1991 ▸; Ruppersberg, 1997 ▸), in addition to other XSA methods modified for ED diffraction (*e.g.* Genzel *et al.*, 2013 ▸), is the basis of data evaluation tools offered on dedicated ED synchrotron beamlines, such as EDDI at BESSY II (up to 2018) and P61A at PETRA III, for depth-resolved residual stress analysis (Apel *et al.*, 2020 ▸).

The present paper addresses another issue related to ED-XSA, which has not been considered in detail so far. The proposed data evaluation strategies exploit two facts:

(i) Depending on the Miller indices *hkl*, the individual ED diffraction lines *E* ^*hkl*^ are affected differently by the material’s inherent elastic (and plastic) anisotropy. This feature was exploited by Daymond & Johnson (2001 ▸) to determine the strain-free lattice parameter *a*_0_ from time-of-flight neutron experiments performed on uniaxially stressed austenitic steel.

(ii) In the near-surface region, which is the transition zone from the biaxial surface to the triaxial volume residual stress state (Hanabusa *et al.*, 1983 ▸; Ruppersberg, 1997 ▸), the average phase homogeneous residual stresses generate *anisotropic* strain fields on the macroscopic scale. It must be emphasized that this kind of macroscopic anisotropy imposed on the material by the residual stress state should not be confused with the macroscopic *elastic* anisotropy present in a single crystal or a material with strong crystallographic texture, which is excluded in the present case. Concepts for XSA on materials featuring a nearly single-crystalline (mosaic) structure were discussed by Hollmann *et al.* (2021 ▸).

**Figure 1:**
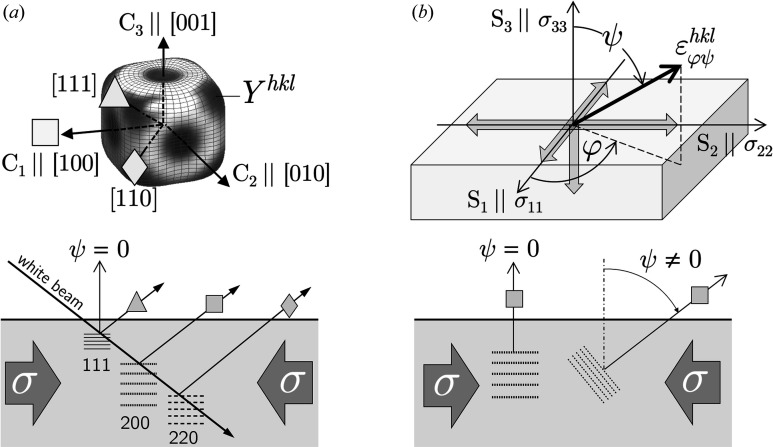
(*a*) Single-crystal (microscopic) and (*b*) stress-imposed (macroscopic) anisotropy in ED-XSA of materials possessing cubic crystal symmetry. {**C**} and {**S**} denote the crystal and the sample reference systems, respectively. *Y* ^*hkl*^ is the directional single-crystal Young modulus (illustrated here using the example of austenitic steel). For further details see text.

The situation is illustrated in Fig. 1 ▸. On the microscopic (*i.e.* crystallite) scale the elastic anisotropy can be represented by the directional Young modulus, *Y* ^*hkl*^, which is given for cubic materials by (Paufler, 1986 ▸) 

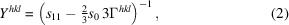

with 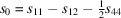 (*s*_*ij*_ are the single-crystal elastic moduli) and the orientation factor 3Γ^*hkl*^ = 3(*h* ^2^ *k* ^2^ + *k* ^2^ *l* ^2^ + *l* ^2^ *h* ^2^)/(*h* ^2^ + *k* ^2^ + *l* ^2^)^2^. Thus, isotropic behaviour corresponds to *s*_0_ ≡ 0, which is equivalent to *A* = 2*c*_44_/(*c*_11_ − *c*_12_) = 2(*s*_11_ − *s*_12_)/*s*_44_ ≡ 1 (*c*_*ij*_ are the single-crystal constants). *A* is the Zener factor, which is used to quantify the single-crystal anisotropy of cubic materials by a single number (Zener, 1948 ▸; Chung & Buessem, 1967 ▸). From Fig. 1 ▸(*a*) it can be seen that, due to the single-crystal elastic anisotropy, different reflections *hkl* in an ED diffraction spectrum will detect different lattice strains ɛ^*hkl*^ within the crystal reference system {**C**}, even for the same measurement direction within the sample reference system {**S**}.

On the macroscopic scale the lattice strain 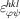 additionally depends on the measurement direction (φ, ψ) in the sample reference system [Fig. 1 ▸(*b*)]. This direction dependency is exploited by those XSA methods which evaluate the stress by regression from strains obtained for various orientations. These include the classical sin^2^ψ method and approaches based on it such as LIBAD (low incidence beam angle diffraction) (van Acker *et al.*, 1994 ▸; Mohrbacher *et al.*, 1996 ▸; Marciszko-Wiackowska *et al.*, 2019 ▸) and the mixed-mode methods (Kumar *et al.*, 2006 ▸; Erbacher *et al.*, 2008 ▸), as well as methods based on two-dimensional X-ray diffraction (2D-XRD) like the cosα method (Sasaki, 2014 ▸; Miyazaki & Sasaki, 2016 ▸) and other 2D-XRD techniques (Keckes *et al.*, 2018 ▸; He, 2018 ▸).

For polycrystalline materials featuring a random crystallographic texture, the experimentally obtained lattice strain 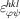 is translated into stress by means of the diffraction elastic constants (DECs) 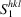 and 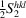 , which are the link between the microscopic and macroscopic scales. For all crystallites which fulfil the Bragg condition within the irradiated sample volume, the DECs determine the degree of the anisotropic (*i.e. hkl*-dependent) deformation in response to the imposed residual stress field. They can be determined experimentally in loading tests or calculated using models that make different assumptions about the grain interaction. The best-known models go back to Voigt (homogeneous deformation in all crystallites; Voigt, 1910 ▸), Reuss (homogeneous stress; Reuss, 1929 ▸), Eshelby/Kröner (elastic polarizability of the crystallites; Eshelby, 1957 ▸; Kröner, 1958 ▸) and Hill/Neerfeld (arithmetic mean of Reuss and Voigt; Neerfeld, 1942 ▸; Hill, 1952 ▸). The quasi-isotropic character of the DECs on the macroscopic scale results from the fact that they do not depend on the measurement direction (φ, ψ) with respect to the sample reference system {**S**}.

In the present paper, the *hkl* dependency of the DECs is used to introduce two methods which allow ED-XSA experiments to be performed under geometric constraints. The paper is structured as follows. In Section 2 the theoretical background of the proposed methods is given and the preconditions for their applicability are defined. Sections 3 and 4 are dedicated to experimental examples from different fields in materials science. The examples are chosen so that the results achieved by the methods introduced here can be compared with those obtained by established techniques such as the sin^2^ψ method. The sample material in this paper serves only as a means to an end to introduce and compare the two XSA methods by practical examples. For detailed material-specific background information on manufacturing and processing, reference is made at the appropriate point to further literature. The discussion in Section 5 is devoted to a critical assessment of the presented methods with respect to their applicability to specific issues of X-ray stress analysis.

## 2. Theoretical background

### 2.1. The extended transverse contraction method

We assume a uniform biaxial residual stress state within the information depth accessible to the X-rays used for measurement, *i.e.* stress depth gradients are assumed to be negligible. The fundamental equation of XSA (Stickforth, 1966 ▸; Evenschor & Hauk, 1975 ▸) then takes the following form: 

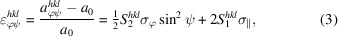

where *a*_0_ is the strain-free lattice parameter for cubic materials and 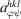 = 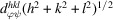 is the *d* spacing normalized to the edge length of the unit cell, *a* ^100^. The stresses 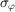 = 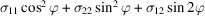 and 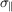 = 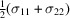 denote the in-plane stress component in the azimuth direction φ and the average in-plane stress, respectively.

**Figure 2:**
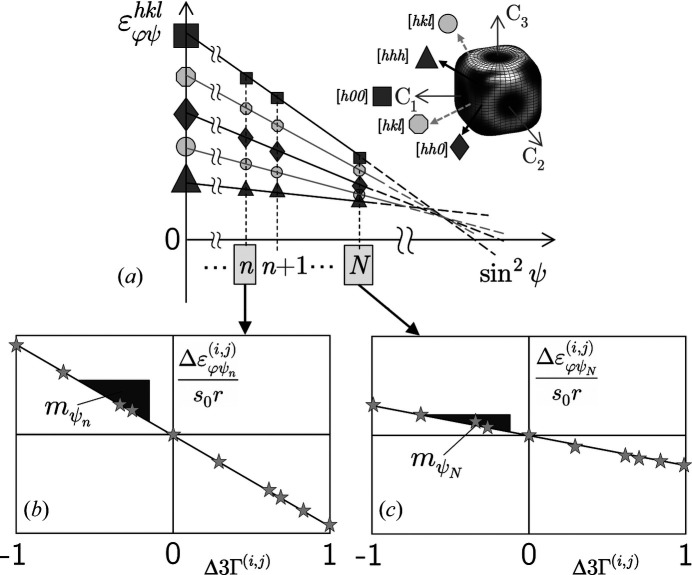
(*a*) The principle of the extended transverse contraction method. The material’s elastic anisotropy is illustrated by the various symbols, which mark different directions in the crystal reference system {**C**} featuring different Young moduli *Y* ^*hkl*^ (calculated for austenitic steel). The slopes of the individual 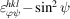 distributions vary within a range limited by the ‘hardest’ (filled triangles) and ‘softest’ (filled squares) crystal directions. (*b*), (*c*) Examples of plots calculated according to equation (6). For further details see text.

The concept of the extended transverse contraction method consists of the evaluation of differences 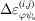 = 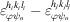 of lattice strains^1^ that were obtained for a series of *P* evaluable reflections *hkl* in the diffraction pattern (*i.e. i*, *j* = 1…*P*) for the same inclination angle ψ_*n*_ (*n* = 1…*N*). Such data sets are available from a sin^2^ψ measurement performed in the ED diffraction mode (Fig. 2 ▸). Due to the single-crystal elastic anisotropy of the material, the 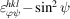 distributions obtained for different reflections *hkl* feature different slopes. Therefore, a sin^2^ψ measurement carried out even for a (strongly) restricted ψ range allows for the evaluation of 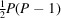 lattice strain differences for any inclination angle ψ_*n*_. This leads to the following system of equations: 

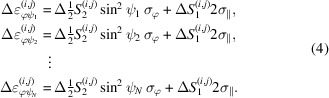

The DEC differences can be written as (see Appendix *A* [App appa]) 

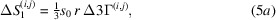




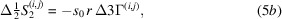

where *r* is the Reuss ratio in the grain interaction model used to calculate the DECs. According to equations (5*a* [Disp-formula fd5a]) and (5*b* [Disp-formula fd5b]), the system of equations (4) can be rewritten as

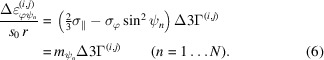

This expression represents a set of *N* linear equations of type *y* = *mx* where *y* contains the lattice strain differences 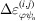 , which may be understood as ‘relative’ strains. In contrast to the ‘absolute’ lattice strain 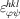 , the ‘relative’ strain is much less sensitive to uncertainties in the strain-free lattice parameter *a*_0_, which can be shown by a Taylor series expansion. Moreover, the stress term in the brackets of equation (6) is not obtained from a single lattice strain difference itself, but from the slope 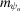 of the regression line fitted to the 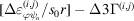 distributions. This justifies the use of a lattice parameter for *a*_0_ which is obtained from averaging all measured lattice spacings. From Figs. 2 ▸(*b*) and 2 ▸(*c*) it can be seen that the slopes 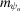 depend on ψ. Note that the five reflections considered in the example result in a total of ten possible combinations for the calculation of strain differences. For the assumptions made (*i.e.* negligible stress gradient), pairs of reflections featuring the same orientation factor 3Γ (*e.g.* 3Γ^111^ = 3Γ^222^ = 1) would result in a strain difference 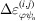 = 0 and consequently these data points should be located at the coordinate origin of the 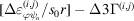 diagram. Significant deviations from this position can have various causes, such as stress gradients or plastic deformation.

For a sin^2^ψ measurement in the azimuth direction φ = 0°, equation (6) takes the form

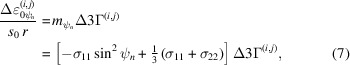

with 

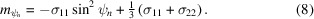

**Figure 3:**
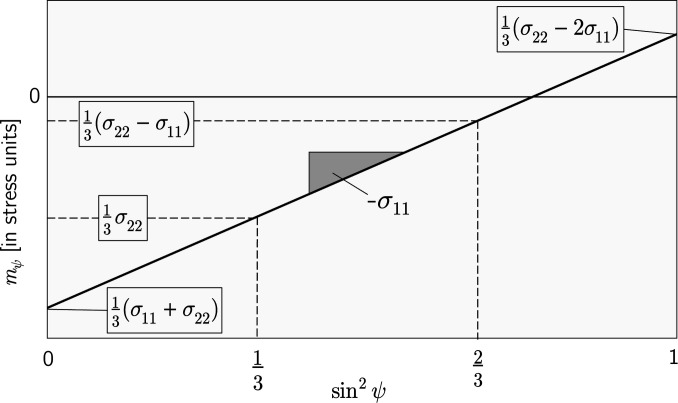
A schematic view of the *m*_ψ_–sin^2^ψ plot according to equation (8). Special values of sin^2^ψ are marked for which *m*_ψ_ yields sums and differences, respectively, of the in-plane stress components.

It can be seen from Fig. 3 ▸ that the *m*_ψ_–sin^2^ψ plot contains some interesting points on the abscissa for which linear combinations of the in-plane stress components can be obtained on the ordinate axis. However, since the approach presented here is aimed at problems where the accessible ψ range is (severely) limited, only points up to about 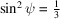 (which would give the stress component σ_22_ in the transverse direction) will be of practical interest. For measurements which can only be performed under ψ = 0°, the method provides the average in-plane stress, *i.e.* 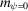 = 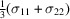 = 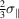 (see Section 4.2).

### 2.2. The optimization method

This approach was theoretically presented by Klaus & Genzel (2019 ▸), but given its complementarity to the method presented in the previous section, the main features will be briefly summarized again here. Taking into account the depth dependence of the residual stress state and following Ruppersberg *et al.* (1989 ▸), equation (3) can be rewritten as

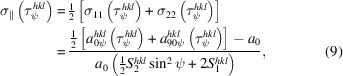

where 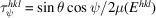 is the information depth for XSA measurements performed in the symmetrical Ψ mode and 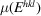 is the energy-dependent absorption coefficient. Equation (9) is of ‘universal’ nature, since its right-hand side contains the pure experimental information (independent of the radiation used and/or reflections *hkl*), whereas the unknown residual stresses are on the left-hand side. Note that the experimentally accessible stresses σ(τ) are usually different from the stresses in real space σ(*z*), due to the exponential attenuation of the X-ray beam by matter. The relationship between them is given by 

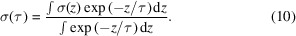

Since this equation has the form of a Laplace transform, the σ(τ) stress depth profiles are called Laplace stresses.

**Figure 4:**
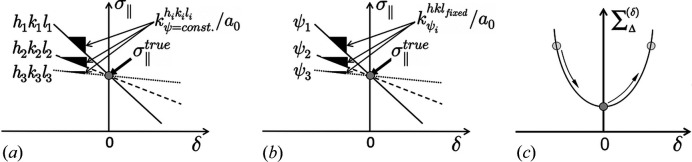
Driving forces in the optimization procedure. Exploitation of (*a*) the microscopic and (*b*) the macroscopic material anisotropy. (*c*) Minimization of the total path length 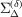 according to equation (12) in the 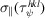 plot (Klaus & Genzel, 2019 ▸). See text for further details.

The fundamental idea of the optimization concept consists of exploiting the strong sensitivity of the lattice strain to variation in the strain-free lattice parameter *a*_0_. We write 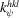 = 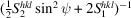 and expand equation (9) into a Taylor series up to the first order regarding an uncertainty δ in *a*_0_ (Klaus & Genzel, 2019 ▸): 

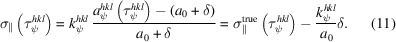




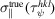
 is the residual stress depth profile evaluated with the actual strain-free lattice parameter *a*_0_. Fig. 4 ▸ shows that each residual stress value 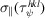 responds differently to changes in *a*_0_ (via δ). This is due to the term 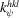 , which causes the slope of the linear equation (11) to depend on *hkl* and ψ on the microscopic and the macroscopic scale, respectively. The point where all straight lines intersect corresponds to the minimum of the path length 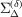 that results when neighbouring points in the discrete 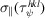 plot are connected,

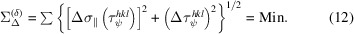

The optimization procedure can be applied even to a data set measured only under ψ = 0°. In this case, however, it provides only the average σ_∥_ of the longitudinal and transverse residual stress components σ_11_ and σ_22_.

### 2.3. The influence of the grain interaction model

Since the approaches introduced in the previous sections exploit the material’s anisotropy, the choice of grain inter­action model used to calculate the DECs 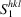 and 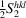 is essential. This applies in particular to the extended transverse contraction method, which is based on the anisotropy term *s*_0_ *r*Δ3Γ^(*i*,*j*)^ introduced in equations (5*a* [Disp-formula fd5a]) and (5*b* [Disp-formula fd5b]).Very recently it was shown that the frequently used Eshelby/Kröner model is not necessarily the appropriate model to describe grain interaction in the material’s near-surface region (Marciszko-Wiackowska *et al.*, 2022 ▸), but should be replaced by a gradient model which takes into account a possible variation from the Eshelby/Kröner model (volume) to the Reuss model or to the direction-dependent ‘free-surface’ model (Baczmanski *et al.*, 2008 ▸) close to the surface.

In the present paper, the DECs are assumed to be constant within the X-ray information depth. The model applied for their calculation is defined by an optimization procedure similar to that described in the previous section. It is also based on minimizing the path length 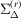 , but here the variable used to find the minimum is the Reuss ratio *r* in the grain interaction model defined by equations (5*a* [Disp-formula fd5a]) and (5*b* [Disp-formula fd5b]), 

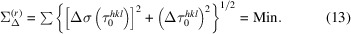

Note that the stress values 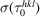 are not obtained from a single strain value [such as the stress values 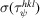 in equation (9)]. Rather they are the average result of a linear regression from the 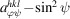 distributions for any reflection *hkl* in the diffraction spectrum which can be assigned to the maximum information depth 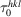 = 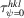 = 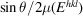 . Klaus & Genzel (2019 ▸) showed that the 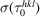 data only form a smooth curve without jumps in Laplace space if the correct grain interaction model is taken for stress evaluation.

## 3. Experimental

### 3.1. Sample material

#### 3.1.1. Superheater tubes

The investigated sample originates from a seamless tube of austenitic stainless steel of type TP347H (ASTM A213), which is commercially applied for superheaters in thermal power plants. The overall chemical composition and a dedicated solution treatment for grain refinement provide good oxidation resistance at high temperatures in steam, which is further promoted by shot peening on the steam side of the tubes. Shot peening on the steam side, thus the inner side, of the tubes is routinely carried out in industry. Although details about the shot-peening process are not disclosed, its influence on microstructure and hardness is clearly evident and has been investigated thoroughly. The beneficial effect of shot peening on reducing steamside oxidation has been revealed both on the laboratory scale mimicking real industrial conditions and after long-term exposure of shot-peened test segments in a Danish thermal power plant (Rosser *et al.*, 2012 ▸; Pantleon *et al.*, 2020 ▸; Kurley & Pint, 2020 ▸).

Seamless superheater tubes with an outer diameter of 33 mm and a wall thickness of 5.6 mm were received, with average grain sizes of about 22 µm in the bulk. The inner surface of the tubes, thus the steam side with concave curvature, was shot peened. Shot peening resulted in nanocrystalline grains at the surface and a microhardness of more than 400 HV, with a corresponding hardness depth profile within a region of about 250 µm beneath the shot-peened surface towards the unaffected bulk with a microhardness of 180 HV. The shot-peened tube was cut into rings of 10 mm thickness, which were further cut into segments for energy-dispersive diffraction on the concave surface, thus the steamside of the superheater tubes. X-ray texture analysis did not reveal a pronounced preferred orientation. Further information on the material’s microstructure is provided by Pantleon *et al.* (2020 ▸).

#### 3.1.2. Weld sample

For the investigation of stress evolution during welding the metastable high-alloy austenitic steel X2CrNi18-9 was used. Detailed information on the chemical composition, the preparatory heat treatment of the sample and the preliminary microstructure investigations is given by Hempel (2022 ▸). Neutron diffraction revealed a negligible rolling texture.

**Figure 5:**
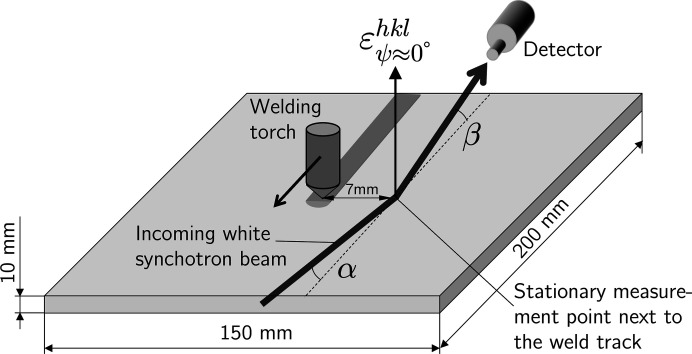
A schematic view of the setup used for the *in situ* welding experiments (not to scale). See text for details.

For welding, sheet metal sections of size 200 × 150 mm were used (Fig. 5 ▸). The heat was applied by a mechanized TIG (tungsten inert gas) welding process without filler metal, *i.e.* the base metal was melted locally. The translational speed of the welding torch was *v*_s_ = 3.33 mm s^−1^. The weld was placed in the centre of the specimen parallel to its long side, with the start and stop positions each 5 mm from the specimen edge. During welding, the specimen was placed on three balls made of ZrO_2_, which allowed defined thermal and mechanical boundary conditions.

### 3.2. X-ray stress analysis

The ED-XSA experiments reported here were carried out some years ago on the materials science synchrotron beamline EDDI at BESSY II. The technical parameters of this beamline, which closed in 2018, can be found in the report by Genzel *et al.* (2007 ▸). The high-energy white photon beam, with a usable energy range between about 8 and 120 keV, was provided by a superconducting 7 T multipole wiggler. The primary beam was confined by slits to a cross section of 1 × 1 mm. The fixed diffraction angle 2θ was chosen as 14° for the shot-peened austenitic superheater tube and 10.25° for the *in situ* welding of austenitic steel. The equatorial divergence of the diffracted beam, which was recorded by a multi-channel germanium detector (Canberra, model GL0110), was confined by double-slit systems with an aperture of 30 µm to values of Δ2θ < 0.01°. The area effectively seen by the detector on the sample surface thus has a lateral extension of about 300 µm.

XSA on the superheater tubes was performed in the symmetrical Ψ mode within a ψ range of 0–60° with a step width of Δψ = 5°. The counting time per spectrum was 180 s. The diffraction angle of 14° was chosen to place a large number (nine) of diffraction lines within the usable energy range provided by the multiple wiggler. The setup for the *in situ* welding experiments is shown in Fig. 5 ▸. The plate was inclined by an angle α = 5° against the incoming beam, and the exit angle between the plate surface and the diffracted beam was defined by β = 5.25° to ensure nearly symmetrical diffraction conditions. The *in situ* experiments were performed under fixed geometric conditions, *i.e.* without any sample tilt or rotation.

The measurements were taken at the mid-length of the weld seam at a lateral distance of 7 mm from the weld centre line (Fig. 5 ▸), *i.e.* in the base material close to the molten zone. The measured diffraction spectra (raw data) were corrected for various effects such as the wiggler spectrum, detector dead time and absorption effects, as well as for background subtraction. The individual diffraction lines were least-squares fitted by pseudo-Voigt functions.

## 4. Results

### 4.1. X-ray stress analysis on the superheater tube material

#### 4.1.1. Conventional analysis and DEC model evaluation

**Figure 6:**
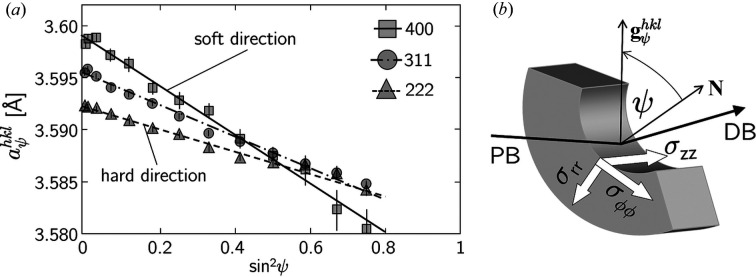
(*a*) Normalized sin^2^ψ distributions obtained for selected reflections *hkl* of austenite. (*b*) A sketch of the diffraction geometry. The designations of the individual stress components take into account the cylindrical specimen symmetry: σ_ϕϕ_, σ_*zz*_ and σ_*rr*_ denote the circumferential, axial and radial stress components, respectively. 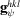 and 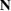 denote the diffraction vector and the surface normal, respectively. PB and DB mark the primary and diffracted beams, respectively.

The segmentation of the tubes allowed the application of the conventional sin^2^ψ method, since tilting the sample along the circumferential direction without beam shading was possible up to ψ = 60°. The results were used as the basis for the validation of the two approaches introduced in this paper. Fig. 6 ▸(*a*) shows almost linear 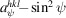 distributions, indicating the absence of steep stress gradients of some hundred megapascals per micrometre that would result in significant curvature for large ψ angles (Klaus *et al.*, 2009 ▸). Furthermore, it is clear that the slope of the regression lines fitted to the data depends on the amount of the Young modulus *Y* ^*hkl*^ in the individual single-crystal directions (Fig. 2 ▸). ‘Hard’ directions (here [111]) offer greater resistance to the stresses than ‘softer’ directions (here [400] and [311]), which is reflected in the flatter slope of the regression line.

**Figure 7:**
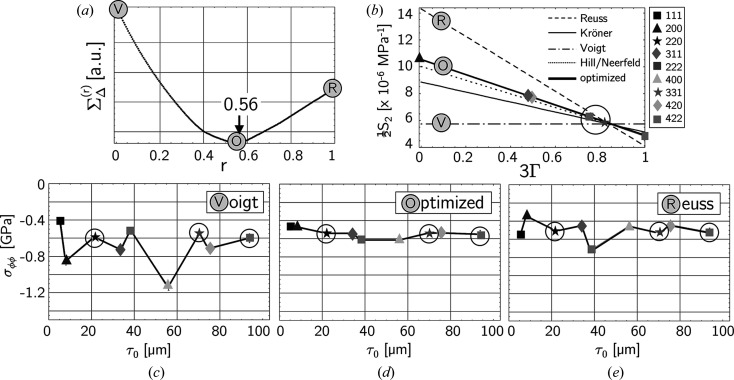
Evaluation of the residual stress depth profile for the circumferential stress component σ_ϕϕ_ and of the grain interaction model. (*a*) Minimization of the total path length 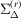 required to connect the individual values 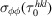 [see equation (13)]. (*b*) DEC 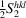 of austenitic steel calculated for different grain interaction models (single-crystal elastic constants taken from Landoldt–Börnstein tables; Hellwege, 1984 ▸). (*c*)–(*e*) Discrete Laplace stress depth profiles calculated for different grain interaction models. See text for further details.

For the evaluation of the sin^2^ψ data the formalism introduced by Klaus & Genzel (2019 ▸) was used (see Fig. 7 ▸ and Section 2.3). The ‘optimized’ DEC model applied to the further analyses in this section was calculated according to equations (15*a* [Disp-formula fd15a]) and (15*b* [Disp-formula fd15b]) using the Reuss ratio *r* = 0.56. Figs. 7 ▸(*c*)–7(*e*) reveal that a smooth residual stress depth profile without (physically unrealistic) jumps is obtained if a grain interaction model is applied which is close to the Hill/Neerfeld model (Hill, 1952 ▸; Neerfeld, 1942 ▸) (arithmetic mean of Reuss and Voigt, *i.e. r* = 0.5). Note that those stress values which are obtained for reflections *hkl* near the model-independent orientation 3Γ* according to equation (17) (circled in the diagrams) hardly change their position on the ordinate axis.

**Figure 8:**
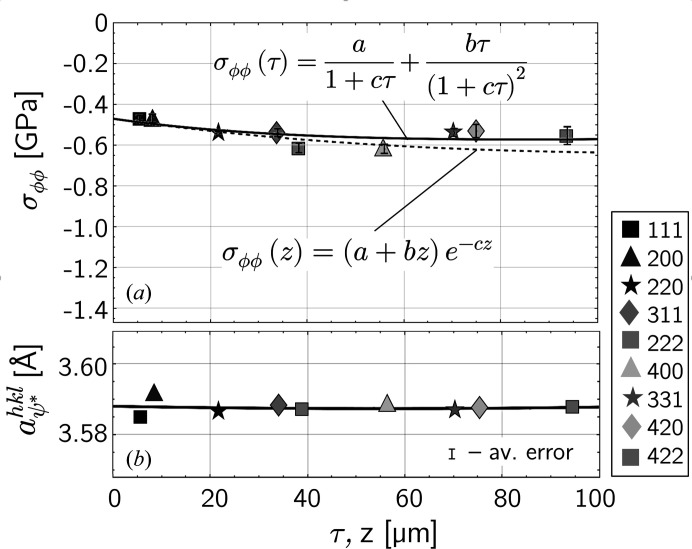
(*a*) Real and Laplace stress depth profiles determined by fitting the σ_ϕϕ_(τ) function to the discrete 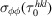 data in Fig. 7 ▸(*d*). (*b*) Normalized lattice parameters obtained from the sin^2^ψ regression lines (Fig. 6 ▸) in the strain-free directions 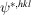 = 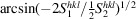 of the biaxial residual stress state, fitted by a linear function.

Concerning the sin^2^ψ analysis performed on samples featuring a cylindrical shape (as in the present case), the near-surface residual stress state must be considered multi-axial, even if it does not depend on the axial and circumferential directions, *i.e.* ∂/∂ϕ = ∂/∂*z* = 0 [note that ϕ, in contrast to φ (azimuth angle in the Cartesian sample coordinate system), denotes the circumferential direction in the cylindrical coordinate system], and if shear stresses are absent, *i.e.* σ_*r*ϕ_ = σ_*z*ϕ_ = σ_*zr*_ = 0. This is due to the remaining coupling term ∂σ_*rr*_/∂*r* + (σ_*rr*_ − σ_ϕϕ_)/*r* = 0 in the differential equilibrium conditions (Timoshenko & Goodier, 1951 ▸). Therefore, the slope of the sin^2^ψ regression line, strictly speaking, yields the stress difference σ_ϕϕ_ − σ_*rr*_ [Fig. 6 ▸(*b*)]. Since we found no evidence for the occurrence of a radial stress component in our experimental investigations (see Fig. 8 ▸), we will confine the considerations in the following to a biaxial stress state, *i.e.* the stress component σ_*rr*_ is omitted in the axis labels.

The discrete 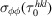 Laplace stress data in Fig. 7 ▸(*d*) can be used to evaluate continuous actual (real space) and Laplace stress depth profiles, σ_ϕϕ_(*z*) and σ_ϕϕ_(τ), respectively. Fig. 8 ▸(*a*) shows an almost uniform compressive residual stress level for the σ_ϕϕ_ component within the information depth of the X-rays, so that the two profiles nearly coincide. The same applies to the lattice parameters 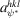 determined from the strain-free direction for the biaxial stress state [Fig. 8 ▸(*b*)]. The absence of a pronounced gradient justifies the assumption of a biaxial residual stress state within the information depth, *i.e.* the presence of the stress component σ_*rr*_ normal to the surface can be excluded. Averaged over all reflections *hkl*, a lattice parameter of 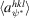 = 3.5877 ± 0.0018 Å results, which can be regarded as the strain-free lattice parameter *a*_0_ under the assumptions made.

#### 4.1.2. Analysis using advanced approaches

In the following, we apply the methods introduced in Sections 2.1 (extended transverse contraction method) and 2.2 (optimization method) to highly reduced 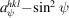 data sets. By limiting the tilt range to ψ ≤ 20° ( 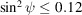 ), it is possible to simulate measurement conditions in samples with complex shapes, where a larger sample tilt would lead to beam shading (*e.g.* measurements in narrow boreholes or at the tooth base of gears). The results achieved in the previous section by means of the conventional well established methods will serve to assess the suitability of the new approaches.

**Figure 9:**
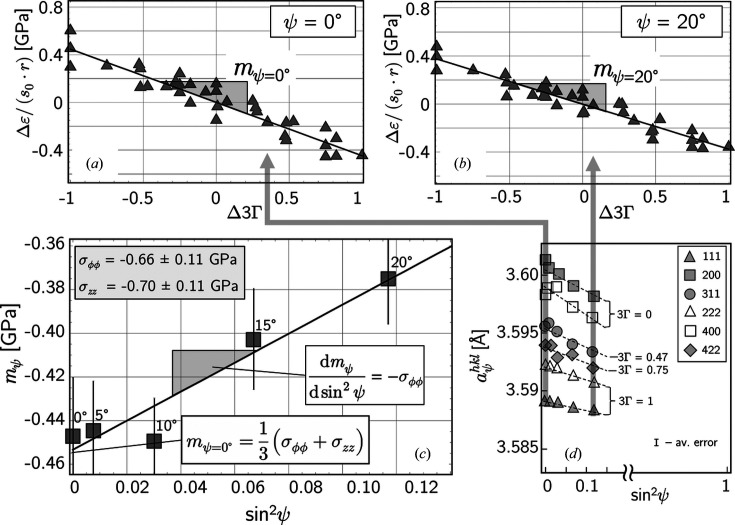
Application of the extended transverse contraction method. (*a*), (*b*) Examples of strain difference plots according to equation (6). The nine diffraction lines taken into account result in 36 pairs of strain differences 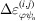 . The error bars are smaller than the plot symbols. (*c*) Plot of the slopes *m*_ψ_ versus sin^2^ψ according to equation (8). (*d*) Reduced sin^2^ψ data set used in the evaluation procedure.

Fig. 9 ▸ depicts the essential steps in the data evaluation concept by means of the extended transverse contraction method, which provides average values for the circumferential and axial stress components, σ_ϕϕ_ and σ_*zz*_, respectively. The value obtained for σ_ϕϕ_ fits well into the residual stress depth profile shown in Fig. 8 ▸. Furthermore, from the fact that the results obtained for both stress components agree within the error margins, it can be concluded that the shot-peening treatment has an in-plane direction-independent effect. Considering only the data measured under ψ = 0° [Fig. 9 ▸(*a*)] does not allow separation of the circumferential and axial stress components, but provides a good approximation for the average in-plane stress.

From the slope 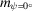 in Fig. 9 ▸(*a*) a value of 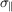 = 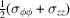 = −0.67 ± 0.04 GPa can be deduced. However, it should be explicitly pointed out that the direct determination of the stress component σ_ϕϕ_ from the slope 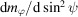 should be treated with caution. From the example shown in Fig. 9 ▸(*c*) the recommendation can be derived to exploit fully the ψ angle range accessible by measurement (cut-off at ψ = 20° in the present case). This area should be covered with as many measurement points as possible to achieve a good statistical validation. If the investigated angular range is too small (in the present case about 10°) the evaluation should be limited to the data obtained under ψ = 0°, which gives the mean value of the longitudinal and transverse stresses.

**Figure 10:**
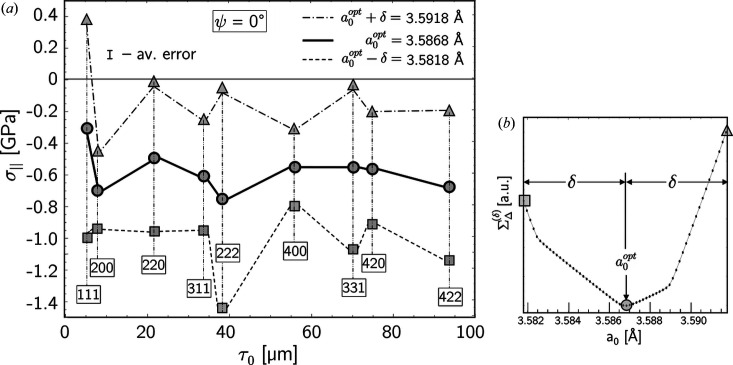
Optimization procedure according to equation (11) applied to the 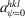 data set [Fig 9 ▸(*d*)]. (*a*) Discrete 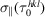 profiles using different values taken for the strain-free lattice parameter. (*b*) The optimized value 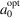 = 3.5868 Å results in the shortest average path length 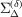 required to connect the individual data points.

The reduced sin^2^ψ data set shown in Fig. 9 ▸(*d*) can be used in a further way that provides a depth profile of the in-plane residual stress state. Based on the universal plot method (Ruppersberg *et al.*, 1989 ▸), the optimization concept introduced by Klaus & Genzel (2019 ▸) (see Section 2.2) represents an extension, in particular by exploiting the high sensitivity of the lattice strains to changes in the strain-free lattice parameter *a*_0_, in order to obtain discrete stress depth distributions in Laplace space with the smallest offset between the individual data points. Fig. 10 ▸ shows that the variation in *a*_0_ leads to both considerable absolute shifts of the 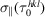 profiles on the ordinate axis and relative shifts between the individual data points in one and the same profile.

**Figure 11:**
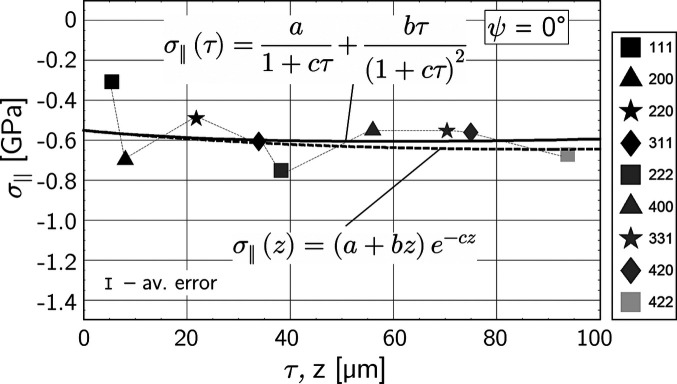
Real and Laplace stress depth profiles determined by fitting the σ_∥_(τ) function to the optimized 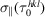 distribution shown in Fig. 10 ▸(*a*). See text for details.

The discrete stress profile obtained for the optimized strain-free lattice parameter 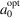 may provide the basis for a least-squares fit to calculate continuous functions for σ_∥_(τ) and σ_∥_(*z*), respectively. The corresponding results are depicted in Fig. 11 ▸ and can be compared with the profiles shown in Fig. 8 ▸. It is noteworthy that, despite the different evaluation histories of the underlying discrete residual stress depth distributions, the two cases yield nearly coincident profiles in both real and Laplace space. The low scatter in the 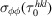 distribution in Fig. 8 ▸ is due to the fact that each individual stress value is the result of a sin^2^ψ regression, which means that scatters of individual strains are averaged out. In contrast, the values in the 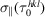 distribution in Fig. 11 ▸ originate from only a single strain 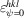 = 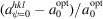 each [*cf.* equation (11)], which explains the much larger scatter. Finally, note that the depth profiles shown in Fig. 8 ▸ are associated with the directional residual stress component σ_ϕϕ_, while the depth profiles in Fig. 11 ▸ correspond to the in-plane residual stress 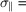 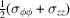 averaged over the circumferential and axial directions. The very good agreement of the depth profiles depicted in Figs. 8 ▸ and 11 ▸ thus confirms the finding gained from Fig. 9 ▸ that the shot-peening process induced an almost direction-independent in-plane residual stress state in the near-surface region of the inner wall of the specimen.

### 4.2. *In situ* study of stress evolution during welding

**Figure 12:**
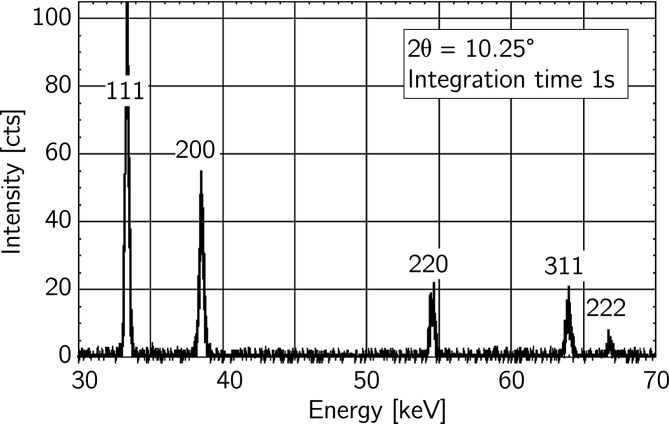
Energy-dispersive diffraction pattern recorded during *in situ* welding of austenitic steel X2CrNi18-9 (Section 3.1.2). Only the part of the spectrum containing evaluable diffraction lines is shown.

The example in this section is to demonstrate that the data evaluation method introduced in Section 2.1 can be applied to study stress evolution during fast *in situ* experiments such as welding. Fig. 12 ▸ shows that the high-flux white synchrotron X-ray beam provided by the 7 T multipole wiggler of the beamline EDDI at BESSY II allowed the acquisition of ED diffraction spectra in a fast sequence of 1 s. The first five diffraction lines were identified as evaluable in further analysis, which results in ten strain differences in the individual 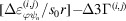 plots.

**Figure 13:**
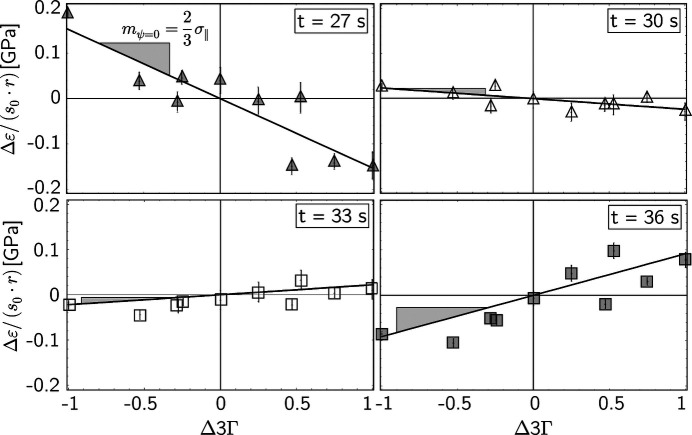
Examples of strain difference plots which represent the average in-plane stress state at different times of the welding process. The individual lattice strains were determined from X-ray diffraction spectra integrated over 3 s each. For the evaluation a Reuss ratio of *r* = 0.64 was applied, which had been obtained by an X-ray load stress analysis to determine the DECs 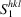 and 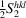 (Hempel, 2022 ▸).

**Figure 14:**
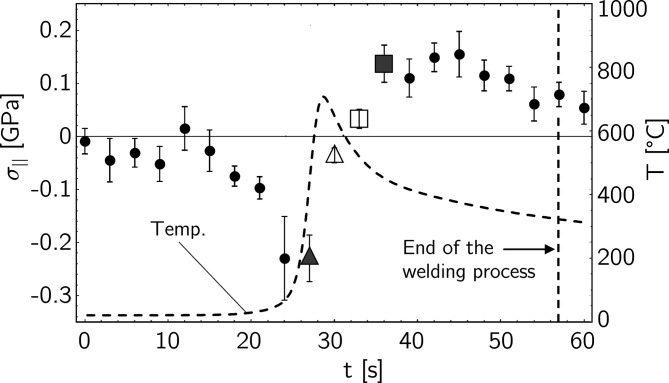
Evolution of the average in-plane stress state during the welding process. Each data point is the result of a regression analysis according to equation (7) for ψ = 0. The points marked by the filled and empty triangles and boxes correspond to the diagrams shown in Fig. 13 ▸. The dashed line depicts the local temporal temperature profile at the measurement point.

The examples in Fig. 13 ▸ reveal a sharp transition from a compressive stress state (indicated by the negative slope of the regression line) to a tensile stress state (positive slope) within a few seconds. The stress evolution during the welding process is shown in Fig. 14 ▸. Starting from an almost residual-stress-free state, a rapid increase in compressive stresses can be observed, reaching their highest level at about the maximum temperature, *i.e.* at the time when the welding torch passes the position next to the measurement point. The temperature then decreases very quickly, which leads to a change in the sign of the stresses within a few seconds (Fig. 13 ▸). The maximum amount of tensile stress generated is slightly less than the amount of the maximum compressive stress before.

Similar to the investigations on the superheater tubes presented in Section 4.1, the results obtained *in situ* in the present case by the transverse contraction method were subsequently verified and confirmed by conventional sin^2^ψ analyses. For this purpose, *ex situ* measurements were performed in the azimuths parallel and perpendicular to the weld seam and then averaged by taking 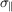 = 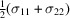 . Additionally, the in-plane stresses σ_∥_ were evaluated by means of the transverse contraction method using the same data, but taking only the lattice strains determined for ψ = 0. The results obtained with these two approaches show good agreement and, moreover, are consistent with the results received from *in situ* measurements for *t* > 1000 s when room temperature was again reached (Hempel, 2022 ▸).

## 5. Discussion

The methods introduced in this paper aim to accomplish X-ray (residual) stress analysis under difficult constraints in terms of accessibility of measurement directions which would be required for the application of conventional sin^2^ψ-based techniques. The experimental examples in the previous section[Sec sec4] have shown that the proposed approaches allow the determination of at least the average in-plane stress state from energy-dispersive measurements performed with ψ = 0. However, their applicability depends on a number of preconditions that must be fulfilled by the material to be investigated.

The restriction to materials with cubic crystal structure is necessary for two reasons:

(i) Normalization of the lattice spacings to one reference parameter *a* ^100^ is required.

(ii) For random crystallographic texture, the elastic material behaviour described by the orientation factor 3Γ^*hkl*^ can be quantified independently of the measurement direction (φ, ψ) in the sample reference system by a single parameter, the Reuss factor *r*, which can be determined by at least one of the proposed approaches (optimization method, Fig. 7 ▸).

Since both methods are based on the evaluation of lattice strains in crystal directions with different elastic behaviour (anisotropy), they require input data in the form of all reflections *hkl* with different 3Γ^*hkl*^ which are evaluable within the diffraction pattern.

Furthermore, both methods should only be applied to materials with pronounced single-crystal elastic anisotropy defined by the Zener factor *A*, such as ferritic (*A* = 2.5) and austenitic steel (*A* = 3.5), copper (*A* = 3.2) or nickel (*A* = 2.6). For materials featuring weak elastic anisotropy such as aluminium (*A* = 1.2) or TiN (*A* = 0.9), its role as a driving force is missing in the extended transverse contraction method (Fig. 2 ▸) and the optimization method [Fig. 4 ▸(*a*)]. However, there is no sharp limit with respect to the Zener factor from which or up to which the methods introduced here are applicable. Since many technical components with complex shapes and hard-to-access measuring points (*e.g.* gears, or parts with boreholes or sharp bends) are made of steel or nickel-based alloys, there are a wide range of applications.

Another requirement that must be met for both methods is the absence of significant nonlinear sin^2^ψ distributions, which can occur as a result of very steep stress gradients, strong plastic deformation or pronounced crystallographic texture. These nonlinearities would disturb the indirectly proportional relation between the magnitude of Young’s modulus *Y* ^*hkl*^ and the increase in the respective sin^2^ψ distribution for the reflection *hkl* [Fig. 6 ▸(*a*)]. The formalisms described in Sections 2.1 and 2.2 would no longer be applicable in this case. For the two examples presented in this paper, this point could be excluded, since the sin^2^ψ measurements performed (for the weld specimens afterwards) had not revealed any non­linearities in this respect.

In general, to exclude such effects, ‘twin experiments’ should be performed on (partially) destroyed samples on which sin^2^ψ measurements are possible. However, it should be noted that cutting the specimen will release some of the residual stresses. Care should also be taken if the methods are to be applied to multi-phase materials. In these cases, the criteria described by Hanabusa *et al.* (1983 ▸) and Ruppersberg (1997 ▸) should be used to estimate whether triaxial residual stress states are to be expected in the depth range covered.

The two methods also differ in terms of some preconditions and the information content, making them complementary for practical applications (see Table 1 ▸). If, as in the case of the superheater tubes (Section 4.1), all preconditions are fulfilled (*i.e.* both stress and composition gradients are negligible), both approaches can be applied and give comparable results with respect to the near-surface residual stress state. In practice, however, more or less pronounced depth gradients of one kind or another will often occur. Since in the case of strongly restricted possibilities for specimen tilting (*e.g.* at the tooth base of gears or in narrow bores) an independent verification (as in Section 4.1) is not possible, it is recommended to evaluate the data with both approaches and then compare the results. This is possible in principle, provided that high-quality data are available in the form of a large number of diffraction lines with sufficient counting statistics that simultaneously reflect the full range of elastic anisotropy by covering ‘hard’ and ‘soft’ crystal directions.

We see two potential areas of application for the methods proposed in this paper. The first area concerns XSA experiments under difficult conditions where conventional sin^2^ψ-based methods fail for geometric reasons, *i.e.* samples with complex shape that cannot be tilted due to beam shadowing. Under such boundary conditions, the proposed evaluation strategies appear, to the best of our knowledge, to be currently the only way of obtaining non-destructive and depth-resolved (optimization method) information on the near-surface residual stress state. However, depending on prior knowledge of the microstructure of the material, the results should be interpreted with due caution. This may mean that in some cases only the sign and magnitude of the near-surface (residual stress) state can be determined in this way. A certain verification may be possible in some cases if the sample is subsequently cut open to expose the measurement point. In this case, however, it must be noted that cutting leads to a partial release of the macro and phase homogeneous residual stresses. Of the examples presented here, this applies to the superheater specimen, where cutting open the tube presumably released some of the residual stresses in the circumferential direction.

A second potential field of application could target the investigation of larger sample series in the sense of an in-process analysis. In this case, the preconditions for the applicability of one or the other (or both) of the methods presented here to the material class to be investigated must be clarified in advance (see Table 1 ▸). The main focus of this application should be the fast determination of relative differences in the near-surface residual stress state, which can occur, for example, due to systematic parameter variation or even random fluctuations in the production process.

## Figures and Tables

**Figure 15:**
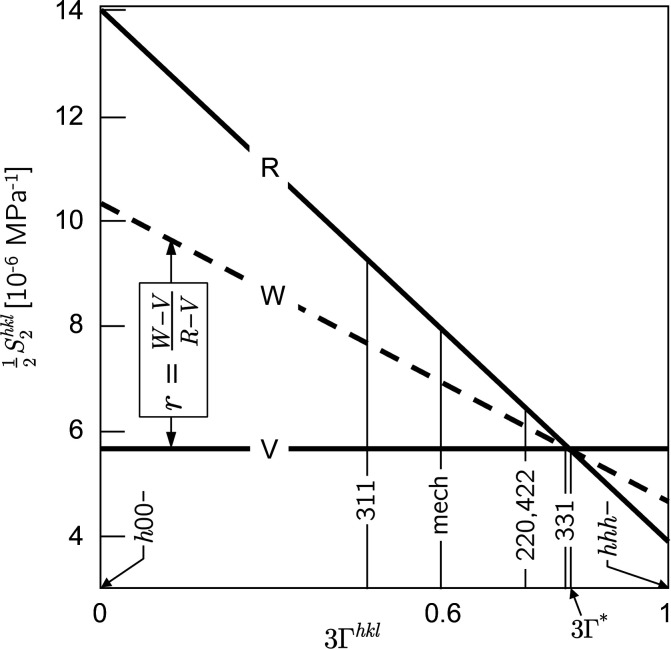
The diffraction elastic constant 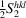 of austenitic steel calculated on the basis of the Voigt (V) and Reuss (R) assumptions. W denotes a weighted model which is an average of Reuss and Voigt with a weighting factor *r*. 3Γ* marks the model-independent orientation and mech denotes the mechanical value, averaged over all orientations.

**Table 1:** Specific features of the methods introduced in this paper

	Extended transverse contraction method	Optimization method
σ(*z*) gradients	Excluded	Allowed
*a*_0_(*z*) gradients	Allowed	Excluded
Output	Average stress within the X-ray information depth	(Residual) stress depth profiles, strain-free lattice parameter

## Footnotes

1 Differences between quantities such as the DECs *S*_1_ and  and the orientation factor 3Γ, which all depend on the Miller indices *h*_*i*_ *k*_*i*_ *l*_*i*_ and *h*_*j*_ *k*_*j*_ *l*_*j*_, are marked in the following by a superscript (*i*, *j*), *i.e.*  =  *etc.*

## Appendix A Diffraction elastic constants

Fig. 15 ▸ depicts the diffraction elastic constant  for austenitic steel, which is used as a sample material for the experimental examples in the present paper. The DECs for cubic materials according to Voigt (V) and Reuss (R) are given by

The weighted model (W) can be expressed by

Taking the difference of the weighted model DECs according to equations (5*a* [Disp-formula fd5a]) and (5*b* [Disp-formula fd5b]), the constant (*i.e. hkl*-independent) terms in the above equations are omitted and we get

Consequently, the differences in DECs no longer depend on the elastic moduli themselves, but become a function of the anisotropy level defined by *s*_0_ and the Reuss fraction *r* (the weighting factor in Fig. 15 ▸) in the model.

The model-independent orientation 3Γ* for cubic materials is given by (Klaus & Genzel, 2019 ▸)

The latter expression is obtained if  is expressed by the elastic moduli *s*_*ij*_. For isotropic materials defined by *s*_0_ ≡ 0 or  =  , one finds 3Γ* = 0.6. This corresponds to the mechanical values  and  which are obtained by averaging not only over the reflecting crystallites but over all orientations.

## References

[bb2] Apel, D., Genzel, M., Meixner, M., Boin, M., Klaus, M. & Genzel, C. (2020). *J. Appl. Cryst.* **53**, 1130–1137. 10.1107/S1600576720005506 PMC7401783 32788906

[bb3] Baczmanski, A., Lipinski, P., Tidu, A., Wierzbanowski, K. & Pathiraj, B. (2008). *J. Appl. Cryst.* **41**, 854–867.

[bb4] Buras, B., Chwaszczewska, J., Szarras, S. & Szmid, Z. (1968). *Fixed-Angle Scattering (FAS) Method for X-ray Crystal Structure Determination.* Report 894/II/PS. Institute of Nuclear Research, Warsaw, Poland.

[bb5] Chung, D. H. & Buessem, W. R. (1967). *J. Appl. Phys.* **38**, 2010–2012.

[bb6] Daymond, M. R. & Johnson, M. W. (2001). *J. Appl. Cryst.* **34**, 263–270.

[bb7] Erbacher, T., Wanner, A., Beck, T. & Vöhringer, O. (2008). *J. Appl. Cryst.* **41**, 377–385.

[bb8] Eshelby, J. D. (1957). *Proc. R. Soc. London Ser. A*, **241**, 376–396.

[bb9] Evenschor, P. D. & Hauk, V. (1975). *Z. Metallkd*. **66**, 167–168.

[bb10] Genzel, C., Denks, I. A., Gibmeier, J., Klaus, M. & Wagener, G. (2007). *Nucl. Instrum. Methods Phys. Res. A*, **578**, 23–33.

[bb11] Genzel, C., Denks, I. A. & Klaus, M. (2013). *Modern Diffraction Methods*, edited by E. J. Mittemeijer & U. Welzel, ch. 5, pp. 127–154. Weinheim: Wiley-VCH.

[bb12] Genzel, C. & Klaus, M. (2017). *Neutrons and Synchrotron Radiation in Engineering Materials Science*, edited by P. Staron, A. Schreyer, H. Clemens & S. Mayer, ch. 9, pp. 161–177. Weinheim: Wiley-VCH.

[bb13] Genzel, C., Meixner, M., Apel, D., Boin, M. & Klaus, M. (2021). *J. Appl. Cryst.* **54**, 32–41. 10.1107/S1600576720014508 PMC7941317 33833639

[bb14] Giessen, B. C. & Gordon, G. E. (1968). *Science*, **159**, 973–975. 10.1126/science.159.3818.973-a 5635993

[bb15] Hanabusa, T., Nishioka, K. & Fujiwara, H. (1983). *Z. Metallkd.* **74**, 307–313.

[bb16] Hauk, V. (1997). *Structural and Residual Stress Analysis by Nondestructive Methods.* Amsterdam: Elsevier.

[bb17] He, B. B. (2018). *Two-Dimensional X-ray Diffraction*, 2nd ed. Chichester: Wiley.

[bb27] Hellwege, K. H. (1984). Editor. *Landoldt–Börnstein Zahlenwerte und Funktionen aus Naturwissenschaften und Technik*, Group III, Vols. 11 and 18. Berlin, Heidelberg, New York: Springer.

[bb18] Hempel, N. (2022). *Zum Einfluss zyklischer Plastizität auf die Eigenspannungsentstehung beim Schweißen hochlegierter Stähle*, Forschungsberichte des Instituts für Füge und Schweißtechnik, Band 61. Düren: Shaker Verlag GmbH.

[bb19] Hill, R. (1952). *Proc. Phys. Soc. A*, **65**, 349–354.

[bb20] Hollmann, A., Meixner, M., Klaus, M. & Genzel, C. (2021). *J. Appl. Cryst.* **54**, 22–31. 10.1107/S1600576720014016 PMC7941319 33833638

[bb21] Keckes, J., Daniel, R., Todt, J., Zalesak, J., Sartory, B., Braun, S., Gluch, J., Rosenthal, M., Burghammer, M., Mitterer, C., Niese, S. & Kubec, A. (2018). *Acta Mater.* **144**, 862–873.

[bb22] Klaus, M. & Genzel, C. (2019). *J. Appl. Cryst.* **52**, 94–105.

[bb23] Klaus, M., Reimers, W. & Genzel, C. (2009). *Adv. X-ray Anal.* **52**, 429–436.

[bb24] Kröner, E. (1958). *Z. Phys.* **151**, 504–518.

[bb25] Kumar, A., Welzel, U. & Mittemeijer, E. J. (2006). *J. Appl. Cryst.* **39**, 633–646.

[bb26] Kurley, J. M. & Pint, B. A. (2020). *Oxid. Met.* **93**, 159–174.

[bb28] Macherauch, E. & Müller, P. (1961). *Z. Angew. Phys.* **13**, 305–312.

[bb29] Marciszko-Wiackowska, M., Oponowicz, A., Baczmanski, A., Braham, C., Watroba, M., Wrobel, M., Klaus, M. & Genzel, C. (2022). *Measurement*, **194**, 111016.

[bb30] Marciszko-Wiąckowska, M., Oponowicz, A., Baczmański, A., Wróbel, M., Braham, C. & Wawszczak, R. (2019). *J. Appl. Cryst.* **52**, 1409–1421. 10.1107/S1600576718004193 PMC5988007 29896059

[bb31] Mittemeijer, E. J. & Welzel, U. (2013). Editors. *Modern Diffraction Methods.* Berlin, Heidelberg: Springer.

[bb32] Miyazaki, T., Fujimoto, Y. & Sasaki, T. (2016). *J. Appl. Cryst.* **49**, 241–249.

[bb33] Mohrbacher, H., Acker, K. V., Blanpain, B., Houtte, P. V. & Celis, J. P. (1996). *J. Mater. Res.* **11**, 1776–1782.

[bb34] Neerfeld, H. (1942). *Mitt. KWI Eisenforsch. Düsseldorf*, **24**, 61–70.

[bb35] Noyan, I. C. & Cohen, J. B. (1987). *Residual Stress Measurement by Diffraction and Interpretation.* New York: Springer.

[bb36] Pantleon, K., Lampert, F. & Montgomery, M. (2020). *Metallography, Microstructure and Analysis*, **9**, 603–614.

[bb37] Paufler, P. (1986). *Physikalische Kristallographie.* Berlin: Akademie-Verlag.

[bb38] Reuss, A. (1929). *Z. Angew. Math. Mech.* **9**, 49–58.

[bb39] Rosser, J. C., Bass, M. I., Cooper, C., Lant, T., Brown, P. D., Connolly, B. J. & Evans, H. E. (2012). *Mater. High Temp.* **29**, 95–106.

[bb40] Ruppersberg, H. (1997). *Mater. Sci. Eng. A*, **224**, 61–68.

[bb41] Ruppersberg, H., Detemple, I. & Krier, J. (1989). *Phys. Status Solidi A*, **116**, 681–687.

[bb42] Ruppersberg, H., Detemple, I. & Krier, J. (1991). *Z. Kristallogr.* **195**, 189–203.

[bb43] Sasaki, T. (2014). *Mater. Sci. Forum*, **783–786**, 2103–2108.

[bb44] Spieß, L., Teichert, G., Schwarzer, R., Behnken, H. & Genzel, C. (2019). *Moderne Röntgenbeugung*, 3rd ed. Wiesbaden: Springer Spektrum.

[bb45] Stickforth, J. (1966). *Techn. Mitt. Krupp Forsch. Ber.* **24**, 89–102.

[bb46] Timoshenko, S. & Goodier, J. N. (1951). *Theory of Elasticity*, 2nd ed. New York: McGraw-Hill.

[bb1] Van Acker, K., De Buyser, L., Celis, J. P. & Van Houtte, P. (1994). *J. Appl. Cryst.* **27**, 56–66.

[bb47] Voigt, W. (1910). *Lehrbuch der Kristallphysik.* Leipzig: Teubner.

[bb48] Webster, P. J., Mills, G., Wang, X. D., Kang, W. P. & Holden, T. M. (1996). *J. Neutron Res.* **3**, 223–240.

[bb49] Withers, P. J. & Webster, P. J. (2001). *Strain*, **37**, 19–33.

[bb50] Zener, C. (1948). *Elasticity and Anelasticity of Metals.* University of Chicago Press.

